# Assessment of Parturition with Cervical Light-Induced Fluorescence and Uterine Electromyography

**DOI:** 10.1155/2013/165913

**Published:** 2013-09-29

**Authors:** Miha Lucovnik, Ruben J. Kuon, Robert E. Garfield

**Affiliations:** ^1^Department of Perinatology, Division of Obstetrics and Gynecology, University Medical Center Ljubljana, Slajmerjeva 3, 1000 Ljubljana, Slovenia; ^2^Department of Obstetrics and Gynecology, University Hospital Heidelberg, Im Neuenheimer Feld 672, 69120 Heidelberg, Germany; ^3^Department of Obstetrics and Gynecology, St. Joseph's Hospital and Medical Center, Downtown Campus at TGen, 445 N 5th Street, Phoenix, AZ 85004, USA

## Abstract

Parturition involves increasing compliance (ripening) of the uterine cervix and activation of the myometrium. These processes take place in a different time frame. Softening and shortening of the cervix starts in midpregnancy, while myometrial activation occurs relatively close to delivery. Methods currently available to clinicians to assess cervical and myometrial changes are subjective and inaccurate, which often causes misjudgments with potentially adverse consequences. The inability to reliably diagnose true preterm labor leads to unnecessary treatments, missed opportunities to improve neonatal outcome, and inherently biased research of treatments. At term, the likelihood of cesarean delivery depends on labor management, which in turn depends on accurate assessments of cervical change and myometrial contractility. Studies from our group and others show that noninvasive measurements of light-induced fluorescence (LIF) of cervical collagen and uterine electromyography (EMG) objectively detect changes in the composition of the cervix and myometrial preparedness to labor and are more reliable than clinical observations alone. We present a conceptual model of parturition constructed on cervical LIF and uterine EMG studies. We also explore how these methodologies could be helpful with managing patients experiencing preterm contractions and with optimizing labor management protocols aimed to reduce cesarean section.

## 1. Introduction

Parturition is a complex process involving increasing compliance of the uterine cervix and activation of the myometrial contractility. Understanding and accurate assessment of these two components are the key to reliable diagnosis and effective management of labor, both at term and preterm. However, methods currently available to evaluate cervical changes and myometrial contractility have several major drawbacks, and evidence shows that misjudgments with important clinical consequences are often made [1–5].

**Figure 1:**
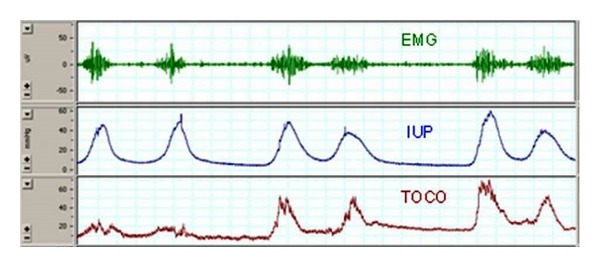
Electrical activity of the myometrium (EMG activity—top trace) is responsible for uterine contractions. Note the excellent temporal correspondence between EMG and mechanical contractile events (measured by intrauterine pressure catheter (IUP), middle trace, and tocography (TOCO), bottom trace).

In our previous studies, we documented evidence that cervical collagen content can be monitored noninvasively by measuring light-induced fluorescence (LIF) of collagen [6]. This method allows assessing the change in cervical structure objectively. Myometrial activity, on the other hand, can be monitored by measuring uterine electromyographic (EMG) activity from the abdominal surface [7–11]. Several studies have demonstrated that uterine EMG detects uterine contractions as reliably as the tocography, and even as the intrauterine pressure catheter (IUP), which is an invasive procedure and cannot be performed anticipating that gestation will be continued (Figure 1) [12–15]. Besides detecting contractions, uterine EMG yields valuable information about changes in the electrical properties of the myometrium which indicate the onset of true labor at term and preterm [7, 9, 16–19].

We present a conceptual model of parturition with a timeline of critical events during labor constructed on data from cervical LIF and uterine EMG studies. We also explore clinical situations, that is, preterm and term contractions, induction of labor, and arrest of labor in the first stage, in which methods to objectively and accurately assess the cervix and the myometrium would be extremely valuable.

## 2. Increasing Cervical Compliance

This process, generally referred to as the “cervical ripening,” summarizes many biochemical and functional changes that result in the softening and effacement of the cervix, allowing cervical dilatation and eventually the delivery of the fetus. During this progressive event, the connective tissue in the cervix, consisting predominantly of collagen, is degraded and rearranged [20]. Cervical ripening does not depend on uterine contractions and is similar to an inflammatory reaction. It involves the infiltration of polymorphonuclear cells and a release of degradative enzymes-metalloproteinases, resulting in a decrease of collagen concentration in the tissue [21]. 

The cervix, its dilation, effacement, consistency, and position are routinely evaluated by digital examination. These are components of the Bishop scoring system, which, although not designed for this purpose, is often used clinically as a predictor of preterm delivery. The clinical exam is, however, a very subjective method to assess the process of cervical ripening [22, 23]. Measuring cervical length by transvaginal ultrasound is more objective and has been shown to have a high negative predictive value for preterm delivery [24–26]. The positive predictive value of cervical length is, however, low, and many patients with a short cervix do not deliver preterm [27].

It has been shown in several studies that changes in collagen content, which are a marker of cervical ripeness, can be assessed non-invasively by measuring LIF of the nonsoluble collagen [28]. This methodology allows an objective assessment of the change in cervical structure, and can detect the change in the composition of the cervix, regardless of its length. It is, therefore, a more accurate method to diagnose cervical ripening.

## 3. Myometrial Contractility

Several events in the myometrium precede labor. Excitability of cells increases due to changes in transduction mechanisms and synthesis of various proteins, including proteins that affect ion channels and receptors for uterotonins [29, 30]. At the same time, systems that inhibit myometrial activity, such as nitric oxide system, are downregulated, leading to withdrawal of uterine relaxation [11]. Electrical coupling between myometrial cells also increases due to an increase in gap junctions, and an electrical syncytium allowing the propagation of action potentials from cell to cell is formed [31, 32]. These changes are required for effective contractions that result in the delivery (expulsion) of the fetus.

The most commonly used method to assess uterine contractions is currently the tocography. Unfortunately, this technique became a standard of care without ever undergoing vigorous clinical trials, 40 years ago, when the standards for clinical evidence were not as rigorous as today. Tocography measures the change in shape of the abdominal wall as a function of uterine contractions and, as a result, is a qualitative rather than quantitative method [33]. It has been shown in several studies that monitoring uterine activity with tocography is not helpful in identifying patients in true (active) labor, both at term and preterm [15, 33–35].

**Figure 2:**
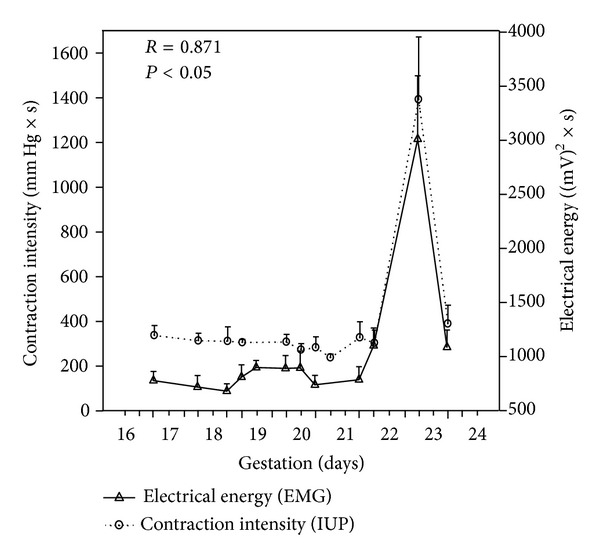
Acute changes in myometrial activity preceding delivery in rats (days 22-23 of gestation). Note the excellent correlation between contraction intensity measured by intrauterine pressure catheter (IUP) and energy of uterine EMG signals. Shi S-Q et al., unpublished data.

The transition from the nonlabor to the labor state of the myometrium can be identified by monitoring the uterine EMG [7, 9, 16–19]. An increase in uterine EMG activity corresponds to the increase of uterine contractility immediately preceding delivery in an animal model (Figure 2). Changes in certain EMG parameters, such as power spectrum (PS) peak frequency and amplitude and propagation velocity of uterine electrical signals, also indicate the onset of true labor at term and preterm in humans [16, 19] (Figure 4(b)).

## 4. Model of Parturition

**Figure 3:**
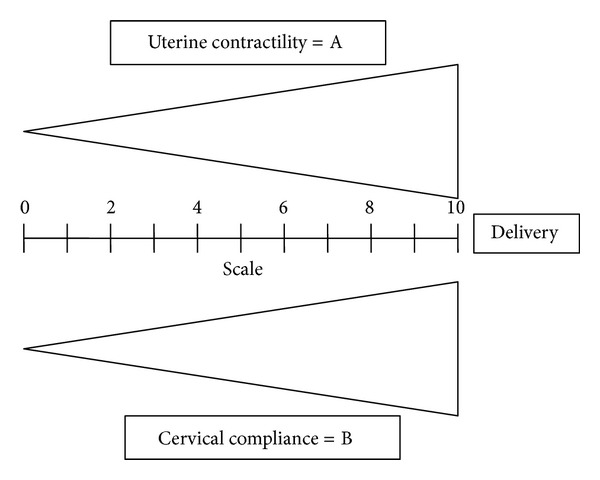
Parturition involves increasing compliance of the uterine cervix and activation of the myometrial contractility.

**Figure 4:**
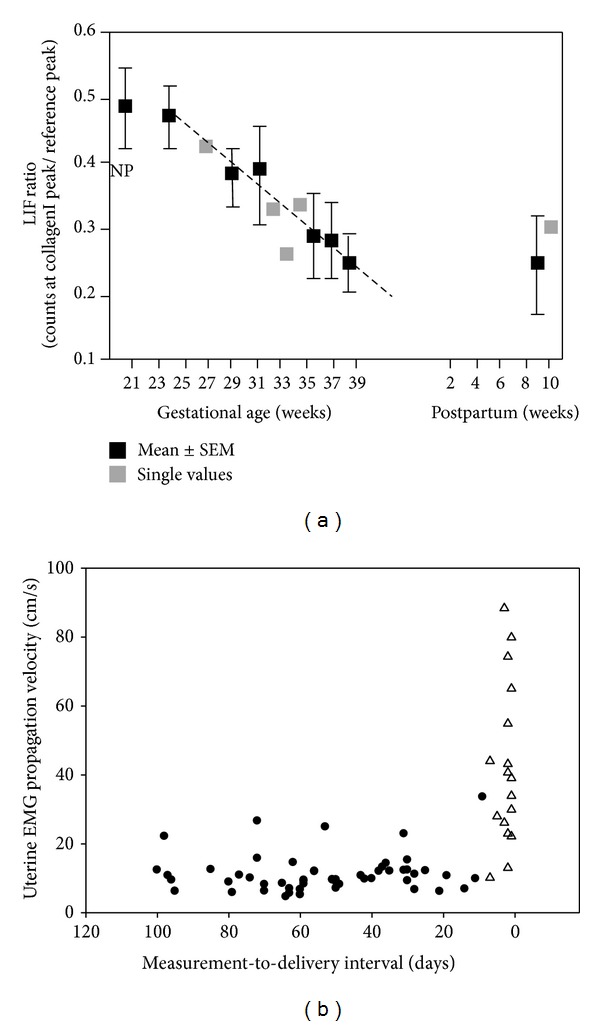
(a) Cervical light-induced fluorescence (LIF) ratio throughout human pregnancy and postpartum. NP: nonpregnant; (b) uterine EMG propagation velocity increases immediately prior to delivery. Δ delivery ≤ 7 days from the measurement; ● delivery > 7 days from the measurement. Based on data from Schlembach et al. [28] and Lucovnik et al. [19].

Figure 3 presents a conceptual model of parturition constructed on data from cervical LIF and uterine EMG studies. The two components of parturition, that is, increasing cervical compliance and activation of the myometrium, take place in a different time frame. According to studies of cervical LIF, and also according to studies of changes in cervical length during pregnancy, the process of softening and shortening of the cervix starts in mid-pregnancy, or even sooner [37, 38] (Figure 4(a)). The myometrial activation, in contrast, is a more acute event, occurring relatively close to delivery. In rats, uterine EMG activity increases not more than 24 hours before delivery (Figure 2). Similarly, in humans the increases of EMG PS peak frequency and propagation velocity, which accurately identify myometrial preparedness for labor, do not typically occur more than seven days from delivery preterm and generally even later at term [16, 19] (Figure 4(b)).

## 5. Diagnosis of Preterm Labor

The inability to reliably diagnose true preterm labor is one of the biggest unsolved problems in obstetrics today. Up to 50% of patients evaluated for preterm labor are not in true labor and will eventually deliver at term [1]. 20% of symptomatic patients, suspected but not confirmed to be in preterm labor, on the other hand, will deliver prematurely [3]. These diagnostic inaccuracies lead to unnecessary treatments, missed opportunities to improve neonatal outcome, and research of potential treatments done on women not chosen on sufficiently objective grounds.

A reliable diagnosis of preterm cervical ripening (increasing compliance) (high B score in Figure 3) and myometrial contractility (high A score in Figure 3) could identify preterm patients who really benefit from early tocolytic therapy, administration of steroids, and admission or transport to a hospital with facilities for neonatal intensive care. It would also help to avoid side effects and substantial economic costs associated with unnecessary treatments. Moreover, it could be extremely valuable in research of potential treatments for preterm labor because it would allow targeting the treatment only to patients who are really in labor [36, 39]. Cervical LIF and uterine EMG, as studied by our groups and others, may be proved in prospective studies to identify increasing compliance and myometrial activation characteristic of preterm labor much more accurately than the methods currently available to physicians today.

**Figure 5:**
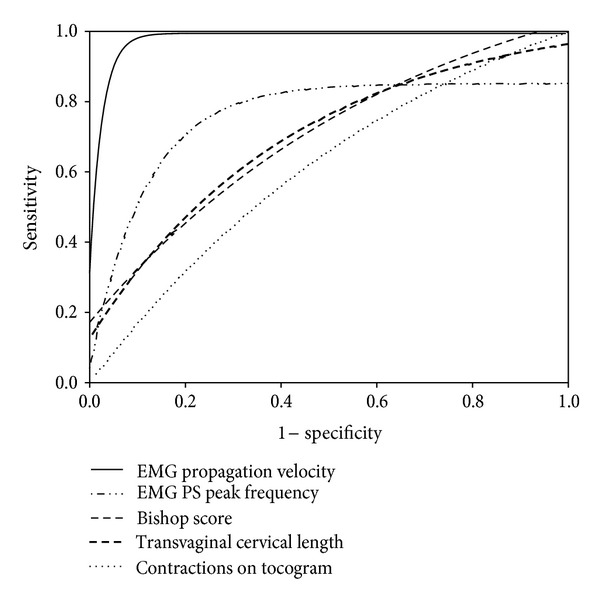
Comparison of receiver-operating-characteristics curves for EMG parameters (power spectrum [PS] peak frequency and propagation velocity) versus currently used methods to predict preterm delivery within 7 days [36].

We have reported a study on 88 patients admitted with the diagnosis of preterm labor at less than 34 weeks of gestational age at a single institution (St. Joseph's Hospital and Medical Center, Department of Obstetrics and Gynecology, Phoenix, Arizona) [19]. Propagation velocity (PV) of EMG signals, power spectrum (PS) peak frequency, and the combination (rescaled sum) of these two parameters were significantly higher in patients delivering within 7 days from the EMG measurement compared to those who delivered after 7 days. Both EMG PV and PS peak frequency identified more accurately the true preterm labor than today's clinical methods (Figure 5). By combining the PV and PS peak frequency, we constructed a model for prediction of sontaneous preterm birth. The area under the receiver-operating characteristics curve for this model was 0.96 (Table 1).

## 6. Lowering Cesarean Delivery Rates

Cesarean section rates have increased significantly worldwide during the last decades but in particular in the middle and high income countries [40–42]. With the growing knowledge of morbidities associated with repeated cesarean sections, many efforts have been made to control this dramatic rise in the rate of cesarean delivery [41, 42]. Given the decline in attempted trials of labor after cesarean, the most effective approach to reducing cesarean section rate is to avoid the first cesarean delivery [43]. Decisions how to induce labor, when to admit patients with contractions, and diagnosis of arrest disorder in the first stage of labor influence significantly the likelihood of cesarean delivery [43]. These management decisions depend heavily on accurate diagnosis of cervical and myometrial changes before and during labor.

A successful vaginal birth is less likely in the absence of a compliant (ripe) cervix. Therefore, accurate assessment of the cervix is crucial in decision-making regarding the method of labor induction. When the cervix is “unfavorable” (low B score in Figure 3), cervical ripening agents should be used since this will increase the chance of vaginal delivery [43]. Cervical LIF can objectively assess the cervical structure [28]. Studies evaluating the usefulness of this technology to decide on using cervical ripening methods should, therefore, be performed. Evaluation of cervical change and myometrial contractility is also important when diagnosing a failed induction, which is defined as failure to generate regular contractions with cervical change [44, 45].

Admission of women in early latent phase of labor has been associated with higher cesarean section rates [46, 47]. Uterine EMG has been shown to identify myometrial activation, characterized by molecular changes leading to an increase in coupling and excitability of cells. This would, therefore, allow clinicians not to admit women in the latent phase or not yet in labor regardless of the presence of contractions on tocogram, since these women are more likely to receive medical intervention such as electronic monitoring, epidural analgesia, oxytocin, and eventually cesarean section [46–48].

Finally, progress in the first stage of labor and/or the diagnosis of arrest in the first stage is based on cervical change and adequacy of contractions. Both of these parameters are difficult to quantify using the currently available subjective methods. On the other hand, cervical LIF can objectively assess change in the cervical structure (score B in Figure 3), and uterine EMG identifies the potential need for additional stimulation of myometrial (score A in Figure 3). 

## Figures and Tables

**Table 1:** Predictive measures of uterine EMG (rescaled sum of power spectrum [PS] peak frequency and propagation velocity) compared with current methods to predict preterm delivery within 7 days [19].

Method	AUC	Best cutoff	Sensitivity	Specificity	PPV	NPV
EMG (PV + PS Peak Frequency)	0.96	84.48	70%	100%	100%	90%
Bishop Score	0.72	10	18%	100%	100%	81%
Transvaginal Cervical Length	0.67	0.7 cm	14%	98%	50%	90%
Contractions on tocogram	0.54	N/A	35%	72%	27%	79%
